# Knee symptoms among college students and their relationship with physical activity behaviors: a cross-sectional survey

**DOI:** 10.3389/fpubh.2026.1879925

**Published:** 2026-07-22

**Authors:** Limin Wang, Peimeng Teng, Qingqing Li, Guijuan He

**Affiliations:** School of Nurisng, Zhejiang Chinese Medical University, Hangzhou, Zhejiang, China

**Keywords:** college student, exercise, knee health, knee symptoms, physical activity

## Abstract

**Objectives:**

To investigate the prevalence of knee symptoms among Chinese college students, and then to analyze the association between physical activities or other factors and knee symptoms.

**Methods:**

Electronic questionnaires on knee symptoms, engagement in physical activity and other variables were administered from June to November 2022. Valid data were analyzed for 1,111 college students. The potential factors associated with knee symptoms were estimated using logistic regression models.

**Results:**

The prevalence of knee symptoms among Chinese college students was 17.37, and 37.44% of students reported knee sounds after prolonged sitting or standing. After adjusting for other factors, moderate-to-high level of vigorous physical activity (odds ratio [OR] = 2.66; 95% confidence interval [CI]: 1.40–5.06; *p* < 0.001) or climbing over 20 floors per day (OR = 2.37; 95% CI: 1.06–5.29; *p* < 0.05) were significantly associated with increased odds of knee symptoms in college students. In addition to knee sounds (OR = 3.54; 95% CI = 2.43–5.16; *p* < 0.001), knee injury (OR = 7.13; 95% CI = 4.30–11.82; *p* < 0.001) had the most influence on the prevalence of knee symptoms.

**Conclusion:**

Targeted interventions should be implemented to improve knee health among college students and promote tailored participation in physical activity.

## Background

Knee osteoarthritis (KOA) is one of the most common chronic diseases associated with disability and represents a substantial and increasing health burden globally (1). The onset and development of KOA are associated with age; the onset always occurs after 40 years of age, and the peak annual incidence is between ages 55 and 64 years (2, 3). The incidence and prevalence of KOA are rapidly increasing with changes in modern environments, and the age of onset is shifting toward younger adults. In the past 20 years, the increased prevalence of knee pain and symptomatic KOA has approximately doubled in women and tripled in men aged 60–74 years in the United States (4). Research from the United Kingdom showed that the incidence of osteoarthritis in individuals aged 35–44 years increased sevenfold from 2003 to 2010 (3), and the prevalence of knee problems among university staff and students aged 18–39 was 31.8% (5). Similarly, a study in Denmark reported a 23% prevalence of knee or hip pain among individuals aged 29–39 years (6). Recent data from China showed that the increase in KOA prevalence was greater among individuals under 35 years of age compared to other age groups, with an annual change of 36.6% (7). Knee health is key to ensuring normal activity to prevent multiple chronic diseases such as hypertension, type 2 diabetes, coronary heart disease, and their complications. From a life-cycle health perspective, physical health in youth is crucial to the health of middle-aged and older adults. Therefore, knee health promotion among adults in their twenties and thirties prior to the onset of KOA has value. Although a mismatch remains between early symptoms and radiographs of the joint (8), particularly in young people who may present with mild symptoms and minimal structural changes, identifying symptoms and preventable factors at an early stage may help to prevent or postpone further structural damage (9). Patient-reported outcomes (PROs) have been emphasized in multiple studies because they can capture important disease-related information prior to the onset of clinical signs or pathophysiological changes (10). Therefore, self-reported knee symptoms are an effective tool to screen for knee problems or ensure early identification of disease (11–14).

Physical inactivity is one of the most common and important modifiable factors related to the incidence and development of KOA and other joint problems. Although physical activity (PA) benefits health in physical, social, and psychological dimensions (15), overload PA is a common factor in knee injury and pain (16, 17). Mueller and Maluf proposed the “Physical Stress Theory” based on how biological tissues adapt to physical stress and concluded that biological tissues exhibit five adaptive responses to physical stress: decreased stress tolerance (e.g., atrophy), maintenance (e.g., homeostasis), increased stress tolerance (e.g., hypertrophy), injury, and death (18). PA is crucial to maintain homeostasis and improve tissue performance for mechanical loading of the knee joint (17, 18). However, the transition to modern modes of transportation and increasingly sedentary lifestyles has substantially reduced overall PA. According to the Physical Stress Theory, two mechanisms may account for the high prevalence of knee injuries and KOA under these conditions. In the absence of physical stress, the joint tissue is in an atrophic and hypofunctional state, with reduced tolerance to mechanical forces. Additionally, insufficient activity also gives rise to glucose and lipid metabolism disorders, leading to an increase in the incidence of overweight and obesity. Excess body weight increases the mechanical load on joint tissues; thus, joint tissues tend to be injured during activities (17, 18). This can lead to acute and chronic joint injuries (17), which are one of the most important factors in the development of KOA (19, 20). Emerging evidence suggests that cumulative fatigue damage resulting from repeated loading may increase the susceptibility of ligamentous tissues to injury, even in the absence of a single traumatic event (21). A study investigating movement coordination after knee injury by Wei et al. demonstrated that persistent alterations in neuromuscular control and motor module organization can influence joint loading patterns during functional activities, such as stair negotiation (22).

The World Health Organization recommends that adults should perform at least 150 min of moderate-intensity aerobic physical activity, or at least 75 min of vigorous-intensity aerobic physical activity, or an equivalent amount of moderate- and vigorous-intensity activity, throughout the week (23). However, the prevalence of insufficient PA is 27.5% globally, and the highest percentage is approximately 60–70% in middle-and high-income countries (24). College students engage in sedentary behavior and exhibit physical inactivity owing to prolonged class attendance and frequent internet use. In Norway, more than 75% of male students and 80% of female students do not meet the recommended physical exercise criteria (25). The physical exercise score of 826 Chinese college students was only 27.4 (±17.72) out of 100 points (26). Evidence indicates that Chinese college students engage in only 2–4 h of physical exercise per week, and spend nearly 40 h per week sitting (27, 28). Moreover, data from a national survey conducted between 1985 and 2014 revealed a significant decline in physical function and exercise ability among Chinese university students (29). Based on Physical Stress Theory, knee symptoms may occur in college students due to decreased stress tolerance in joints resulting from inactivity. To the best of our knowledge, only Ibeachu et al. has previously reported knee problems among college students with a small number of staff taking part (5). In the present study, our objective was to investigate the prevalence of knee symptoms among Chinese college students and to analyze the influencing factors, especially their relationship with physical activity. Based on Physical Stress Theory, we hypothesized that high-level physical activity was associated with greater odds of knee symptoms among college students.

## Materials and methods

### Design

In this study, a cross-sectional design was used.

### Participants

We mainly used convenience sampling from five universities in Zhejiang Province and Beijing, China. Recruitment was conducted via advertisements placed on noticeboards or via electronic posters distributed through student groups. Students who were interested in participation could completed an electronic questionnaire. Additionally, all participants were allowed to share the questionnaire link with their friends or classmates. To avoid potential selection bias, where symptomatic individuals might be more likely to complete the questionnaire than asymptomatic individuals, we designed the name of the questionnaire to emphasize its focus on PA characteristics without targeting any specific level of physical activity. The inclusion criteria for participants were as follows: (i) students aged ≥18 years old; (ii) students enrolled across any college nationally. The exclusion criteria were as follows: participants suffered from an acute knee injury within the past 6 months or had any surgery which was recommended inactivity by doctors. The questionnaire data were collected between June and November 2022. The method of participant sampling has been reported in detail previously (30).

### Sample size

Sample size was estimated using the Power Analysis and Sample Size software (PASS 2008, NCSS Corporation). Based on a logistic regression model with a binary outcome, an *α* of 0.05, and a *β* of 0.10, the required sample size was 1,060. The estimate was adjusted to account for a multiple regression of the independent variable of interest on the remaining independent variables in the logistic regression model, assuming an *R*^2^ of 0.10. Given the non-response rate of 5%, the target sample size was increased to 1,113.

### Variables and instruments

Demographic characteristics of college students, including sex, age, and school year, were collected. Body mass index (BMI) was calculated using height and weight and was divided into underweight (<18.50 kg/m^2^), normal (18.50–23.99 kg/m^2^), overweight (24.00–27.99 kg/m^2^), and obese (≥ 28.0 kg/m^2^) according to recommended classifications for Chinese individuals (31).

### Knee symptoms, sounds, history of injury

Knee symptoms, sounds, and injury were assessed using the same or similar questions widely used in previous studies (6, 12, 32, 33). A self-reported chronic and frequent knee symptom was defined in this study, which was evaluated using the following question: “Have you often (i.e., three or more times per week) experienced pain, stiffness, or limitation of activity in or around the knees in the past 6 months?,” which was adapted from the item developed by O’Reilly et al. to evaluate the prevalence of knee pain in the population (11). Similar questions have been widely employed in studies involving participants with early-stage KOA (12–14). Knee sounds were evaluated with the question: “Are you often (i.e., three or more times per week) disturbed by knee sounds after joint movement when you sit or stand for a long time?” The history of knee injury was evaluated by “Have your knees been injured so that it resulted in limited walking for at least 7 days? “The participants answered: “Neither” “Yes, only the left,” “Yes, only the right,” or “Yes, both.” Those who responded with any affirmative answer were considered to have knee symptoms, sounds, or injuries. Before conducting the survey, we invited two clinicians and professors in the field of osteoarthritis to assess whether the three questions were appropriate in practice, and subsequently modified the questions based on their comments, confirming the face validity. We also invited 40 students to the pre-test to verify the suitability, readability, and retest reliability within 2 weeks. The test–retest reliability, assessed by unweighted Kappa (*κ*), was 0.98, 0.72, and 0.81 for the three questions, respectively.

### Physical activity

The original questionnaire of PA in this study is a revised version of the International Physical Activity Questionnaire Short Form (IPAQ-SF). Participation in vigorous/moderate/light physical activity, including its frequency and duration, was also investigated. The participants were asked whether they engaged in vigorous physical activity (VPA), moderate physical activity (MPA), or light physical activity (LPA) for at least 10 continuous min in a typical week. VPA refers to activities that take considerable physical effort and make breathing effort much harder than normal (such as heavy lifting, digging, aerobics, fast bicycling, or cycling with a heavy load); MPA refers to activities that take moderate physical effort and can result in breathing effort somewhat harder than normal (e.g., carrying light loads, bicycling at a regular pace, mopping the floor); and LPA mainly refers to walking, including walking at work, traveling from one place to another, and walking for recreation. Participants who responded affirmatively were asked to report how many days they participated in each type of activity and how much time they spent participating in each time (<30 min, <2 h, <4 h, ≥4 h). Referring to the Physical Activity Rating Scale-3 (34), we first recoded the duration (coded as 1–4) and frequency (1–2 days, 3–5 days, and 6–7 days coded as 1–3, respectively) of PA. Because the association between PA and KOA or knee symptoms has been inconsistent to date due to variations in intensity, duration, and frequency, this study mainly aimed to explore the associations between LPA/MPA/VPA combination of duration and frequency with knee symptoms. Referring to the methodology of PA score and rating (34, 35), VPA/MPA/LPA score was calculated by multiplying the duration by the frequency, and then referring to the cutoffs of PARS-3, which considered 20% and 40% of full score as the cutoffs of low, moderate, and high levels. PA scores were classified as follows: no PA (0 points), low PA (1–4 points), and moderate-to-high PA (5–12 points). The test–retest reliability of the PA scoring system, assessed by Pearson’s correlation coefficients, was 0.80 for VPA score, 0.89 for MPA score, and 0.84 for LPA score in this study. Supplementary sensitivity analyses for the PA score system using alternative categorizations yielded similar significant results (presented in Supplementary material), but model fit indices supported that the present cutoff values provided a better fit. To better understand the characteristics of PA among college students, we also collected data on daily activities, including average steps, number of climbing floors, and hours of sitting per day, in the last 3 months.

### Statistical analysis

The present study followed the STROBE checklist for cross-sectional designs. Data was analyzed using STATA version 15.0 (Stata Statistical Software, Stata Corp LLC). Descriptive statistics [such as mean and standard deviation (SD), frequencies, and percentages] were calculated to indicate demographic characteristics. Owing to the binary dependent variable, we conducted univariable and multivariable logistic regression models to analyze factors associated with knee symptoms, including age, sex, BMI, knee sound, history of knee injury, PA scores, and steps, climbing floors, and sitting time per day. To mitigate common method bias (CMB), we assured participants of data anonymity, confidentiality, and exclusive use for this study; emphasized the importance of their responses for advancing related research; and simplified questionnaire items and response options to reduce completion time. These procedures are consistent with recommendations by Podsakoff et al. (36). Testing indicated no multicollinearity between dependent variables with variance inflation factors (VIF) ranging from 1.06 to 1.55; although no established method exists for testing CMB, Kock considered VIFs ≤ 3.3 as evidence of CMB-free models (37). All candidate variables were first evaluated via unconditional univariable logistic regression analysis. The variables were subsequently selected into multivariable logistic regression analyses while considering statistical significance and the value in clinical practice. To select a subset of variables while reducing the risk of overfitting (38), in the multivariable logistic regression analysis, stepwise selection (with entry criterion *p*-value equal to 0.05 and removal criterion *p*-value equal to 0.05) was used via 1,000-replication bootstrapping to obtain stable and unbiased parameters. Odds ratios (ORs) and 95% confidence intervals (CIs) were also determined. A *p*-value of ≤ 0.05 (two-sided) was considered significant.

### Ethical considerations

Ethical approval was obtained from A University Biomedical Ethics Committee in June 2022 (IRB20220607-8). All participants voluntarily participated in the study, and since participants were adults (≥18 years), informed consent for participation in this study was directly obtained from the participants themselves, not their legal guardians. The data collected was anonymized, kept confidential, and used exclusively for the present study.

## Results

### Participant characteristics

A total of 1,123 questionnaires were completed, and 1,111 were considered valid after double-checking the responses. Among the 12 invalid questionnaires, seven were excluded because of missing or incorrect age (e.g., aged under 15 or over 40 years). Since age is a key factor associated with knee symptoms and may act as a confounder in the association with PA, we removed these cases to minimize potential bias. The remaining five were excluded because of lacking school names, which made it impossible to ascertain their student status in line with the inclusion criteria. Of the 1,111 students included, 783 (70.5%) were recruited from Zhejiang and Beijing, while the remaining 328 were from schools in other provinces.

The descriptive characteristics of the 1,111 participants and their daily activities are presented in Table 1. The mean age was 20.86 years (±2.63), and 720 participants were women (64.81%). Among the 1,111 participants, the prevalence of knee symptoms was 17.37, and 37.44% experienced knee sounds after joint movement while sitting or standing for an extended time. The mean number of steps per day was 7,409 (±2,533), the number of sitting hours per day was 6.37 (±1.99), and the quartiles of climbing floors per day were 5, 6, and 8 in P_25_, P_50_, and P_75_, respectively. Most college students walked between 7,000 and 10,000 steps (38.14% of the sample), climbed 6–10 floors (35.38%), and sat for 5–8 h every day (43.60%).

**Table 1:** The demographic characteristics, knee symptoms and daily life activities of participants.

Characteristic	Mean (SD)/*n* (%)
Age (years)	20.86 (2.63)
Sex	
Male	391 (35.19)
Female	720 (64.81)
BMI level	
Normal weight	311 (27.99)
Under weight	628 (56.53)
Overweight	129 (11.61)
Obese	43 (3.87)
Knee symptoms	
No	918 (82.63)
Yes	193 (17.37)
Knee sounds	
No	695 (62.56)
Yes	416 (37.44)
Knee injury	
No	995 (89.56)
Yes	116 (10.44)
Steps per day	
<3,000	175 (15.78)
3,000–4,999	213 (19.21)
5,000–6,999	147 (13.26)
7,000–10,000	423 (38.14)
>10,000	151 (13.62)
Climbing floors per day	
<5	437 (39.55)
6–10	391 (35.38)
11–15	110 (9.95)
16–20	115 (10.41)
>20	52 (4.71)
Sitting hours/day (h)	
<5	432 (38.92)
5–8	484 (43.60)
8–10	121 (10.90)
>10	73 (6.58)

BMI, body mass index; LPA, light physical activity; MPA, moderate physical activity; PA, physical activity; VPA, vigorous physical activity.

### Engagement in physical activity

Only 52.93% of respondents commonly engaged in VPA; among them, 47.45% engaged in VPA 3–5 days a week and 40.82% engaged in VPA 1–2 days a week. The duration of the VPA for most students (45.07%) was less than 30 min and 80.44% had a low VPA score. The percentage of students who engaged in moderate and light PA was relatively high. Performing MPA or LPA 3–5 times a week was the most common frequency. A total of 52.62% of students participated in MPA for less than 30 min, while the percentages of students participating in LPA for less than 30 min (38.05%) or 30–60 min (39.35%) were comparable. Although most of the students participated in MPA or LPA at a low level, the percentage of students with moderate-to-high level of LPA was much higher (35.57%). Other characteristics of PA engagement are summarized in Table 2.

**Table 2:** The characteristics of engagement in VPA/MPA/LPA [*N* = 1,111, *n* (%)].

Characteristics	VPA	MPA	LPA
Not engaged	523 (47.07)	349 (31.41)	186 (16.74)
Yes	588 (52.93)	762 (68.59)	925 (83.26)
Frequency (days/week)			
1–2	240 (40.82)	267 (35.04)	210 (22.70)
3–5	279 (47.45)	368 (48.29)	363 (39.24)
6–7	69 (11.73)	127 (16.67)	352 (38.05)
Duration (minutes)			
<30	265 (45.07)	401 (52.62)	352 (38.05)
30–60	202 (34.35)	256 (33.60)	364 (39.35)
60–120	96 (16.33)	65 (8.53)	125 (13.51)
>120	25 (4.25)	40 (5.25)	84 (9.08)
Physical activity score			
Low level	473 (80.44)	634 (83.20)	596 (64.43)
Middle-to-high level	115 (19.56)	128 (16.80)	329 (35.57)

LPA, light physical activity; MPA, moderate physical activity; PA, physical activity; VPA, vigorous physical activity.

### Univariable and multivariable analysis

Table 3 shows the results of the univariable and multivariable logistic analyses. Although BMI was not significantly associated with the prevalence of knee symptoms in the univariable logistic analysis, considering the important effects of metabolism and body weight on the knee joint, we included BMI in the multivariable model. The Hosmer–Lemeshow goodness-of-fit test suggested adequate model fit (*χ*^2^ = 6.27, *p* = 0.62). The Nagelkerke pseudo-*R*^2^ was 0.17, and the corresponding Akaike information criterion (AIC) and Bayesian information criterion (BIC) values were 845.72 and 930.63, respectively. Age, knee sounds, history of knee injury, engagement in moderate-to-high level of VPA, and climbing over 20 floors per day were significantly associated with the odds of knee symptoms. Knee sounds (OR *=* 3.54; 95% CI = 2.43–5.16; *p* < 0.001) and knee injury (OR *=* 7.13; 95% CI *=* 4.30–11.82; *p* < 0.001) had the most influence on the prevalence of knee symptoms after adjusting other factors.

**Table 3:** Logistic regression models for correlated factors of knee symptoms.

Predictors	Univariable OR (95% CI)	Multivariable OR (95% CI)
Age (years)	1.07 (1.01–1.13)^*^	1.08 (1.01–1.15)^*^
Female	0.78 (0.57–1.08)	—
BMI level		
Normal weight	Reference	Reference
Under weight	0.88 (0.61–1.27)	1.14 (0.75–1.75)
Overweight	1.13 (0.70–1.83)	1.02 (0.55–1.90)
Obese	1.25 (0.58–2.67)	0.93 (0.36–2.39)
Knee sounds	4.56 (3.27–6.35)^***^	3.54 (2.43–5.16)^***^
Knee injury	10.31 (6.80–15.63)^***^	7.13 (4.30–11.82)^***^
Steps per day		
<3,000	Reference	—
3,000–4,999	0.67 (0.38–1.19)	—
5,000–6,999	0.93 (0.51–1.70)	—
7,000–10,000	1.17 (0.74–1.87)	—
>10,000	1.58 (0.91–2.72)	—
Climbing floors per day		
<5	Reference	Reference
6–10	1.18 (0.82–1.71)	1.03 (0.67–1.60)
11–15	1.15 (0.66–2.02)	1.23 (0.64–2.36)
16–20	1.31 (0.77–2.23)	1.26 (0.64–2.49)
>20	2.24 (1.16–4.31)^*^	2.37 (1.06–5.29)^*^
Sitting hours per day (h)		
<5	Reference	—
5–8	1.34 (0.95–1.89)	—
8–10	0.85 (0.47–1.52)	—
>10	1.68 (0.92–3.08)	—
Physical activity score^§^		
VPA score		
Low level	1.50 (1.07–2.12)^*^	1.31 (0.85–2.01)
Middle-to-high level	3.00 (1.88–4.79)^***^	2.66 (1.40–5.06)^***^
MPA score		
Low level	1.17 (0.82–1.68)	0.82 (0.49–1.38)
Middle-to-high level	1.98 (1.21–3.25)^**^	0.74 (0.34–1.59)
LPA score		
Low level	0.98 (0.63–1.53)	—
Middle-to-high level	1.21 (0.75–1.94)	—

^*^p ≤ 0.05, ^**^p < 0.01, ^***^p < 0.001; ^§^For PA scores analysis, no PA was set as reference.

BMI, body mass index; CI, confidence interval; LPA, light physical activity; MPA, moderate physical activity; OR, odds ratio; PA, physical activity; VPA, vigorous physical activity.

## Discussion

Although adults rarely experience KOA in their 20s, attention should be paid to early symptoms and knee health in young adults due to the rapid growth of knee problems in young individuals. Our study showed that the prevalence of knee symptoms among Chinese college students was 17.37%, and in this group, 37.44% experienced knee sounds after sitting or standing for a prolonged period.

Increased longevity and a high prevalence of obesity are considered to be the main factors contributing to the increase in the prevalence of KOA; however, Wallace et al. (39) analyzed knee eburnation of the complete skeletons of early industrial individuals (*n* = 1,581), postindustrial individuals (*n* = 819), and hunter-gatherers or farmers of the prehistoric period (*n* = 176). The results showed that the prevalence of KOA in the postindustrial sample was 2.6 times higher than that of the early industrial sample and 2 times higher than that of the prehistoric sample (39). Even after controlling for age and BMI, the prevalence of KOA increased twofold from the early industrial to the postindustrial period (39). Furthermore, among individuals with KOA, the proportion of bilateral cases of KOA in the postindustrial sample (42%) was 1.4-fold higher than that in the early industrial sample (30%) and 2.5-fold higher than that in the prehistoric sample (17%) (39). Ericsson et al. followed 1,064 women aged 25 at baseline for10 years and reported that 33% of them experienced knee pain (40). The present study confirms the high prevalence of knee symptoms in young college students. As a mismatched disease in modern environments (39, 41), factors related to knee problems must be identified to prevent its increasing prevalence and tendency in younger adults.

Physical inactivity has been an important lifestyle change from prehistoric to modern life, and sedentary lifestyles are common worldwide (24). Physical stress during PA is necessary for maintaining homeostasis and improving joint tissue performance (17, 18). Moreover, based on the on the Physical Stress Theory (18), there is a theoretically idealized level of PA that would provide sufficient physical stress to maintain homeostasis or increase the stress tolerance of the knee joint, whereas PA above or below that level would directly or indirectly injure the joint. Although experts in bone and joint health research recommend that physical exercise and activities should be individually adapted to joints to prevent KOA and optimize knee health (42), few studies to date have clarified which specific PA characteristics benefit individual knee health, including PA type, intensity, frequency, duration, and total volume. Our study showed that moderate-to-high level of VPA and climbing over 20 floors per day were significantly associated with odds of knee symptoms among college students. This result is, to a certain extent, consistent with those of previous studies (12, 43, 44). Research has shown that individuals who run 20 miles or more per week have a twofold increase in the risk of symptomatic KOA (44). The findings of the Framingham Heart Study (43) showed that engaging in VPA for more than 2 h per day would increase the risk of symptomatic KOA (OR = 5.3, 95% CI: 1.2–24) and the effect was also significant for radiographic KOA (OR = 1.3 per h, 95% CI: 1.1–1.6). A recent study from the Osteoarthritis Initiative reported that lower physical activity (PASE score of 100–140) or higher physical activity scores (PASE score > 220) significantly increased the risk of joint space narrowing, with ORs of 1.73 and 1.83, respectively (16). Based on the Physical Stress Theory, when engaging in excessive high-level PA in the long term, a cumulative load can cause tissue fatigue, the tolerance to load might be reduced, and injury of the joint may occur due to a chronic or acute overload stimulus (18). Two mechanisms can explain why people tend to experience injury during PA in modern environments. First, when individuals maintain a sedentary and inactive lifestyle over the long term, their joint tissues adapt to the underloading environment, and their tolerance to physical load decreases. Second, under sedentary and physically inactive lifestyles, people tend to become overweight, and higher body weight increases the physical load during physical activity and exercise. Any knee injury, whether from a chronic or acute mechanism, may accelerate the knee symptoms or structural damage of the joint. Hence, research on PA tailored to knee health should be conducted in the future to avoid the harm of overload or underload. Considering the inconsistent effects of VPA, MPA, and LPA on the prevalence and incidence of KOA owing to variations in assessment methods, activity categories, and populations (45), more precise devices to accurately assess PA over an extended period with consistent methods of PA classification should be developed.

In the present study, after adjusting for other factors, college students with a history of knee injury had 7.13 times greater odds of experiencing knee symptoms compared with those without such a history. Previous studies have shown that knee injury is an important risk factor for KOA, especially in the long term. The 10-year incidence of KOA after knee injury (e.g., anterior cruciate ligament tear) was 74%–80% (19, 20). The synthesized evidence from Poulsen et al. (46) showed that injuries to the anterior cruciate ligament or meniscus increased the risk of KOA by 4.2-fold (OR = 4.2, 95% CI 2.2–8.0) and 6.3-fold (OR = 6.3, 95% CI 3.8–10.5), respectively. Hence, maintaining and improving knee joint performance and tolerance to physical load is important for preventing knee injury and reducing the prevalence of KOA. Although knee sounds were correlated with the odds of knee symptoms among college students in this study (OR = 3.54, *p* < 0.001), this association could represent a potential overestimation of pathological significance to some extent. Cibere et al. reported that the reliability coefficient between general crepitus after active movement and KOA was 0.78 in unstandardized clinical practice (33), indicating that self-reported knee noise can, to some extent, serve as an indicator of early KOA. However, with the mean age of participants in this study of 20.86 years and the lack of imaging verification, these knee sounds may represent benign physiological crepitus (47). Although knee sounds were included as a covariate (rather than part of the outcome) because they represent a distinct self-reported phenomenon, a potential overlap in symptom perception might exist.

While the proportion of college students engaged in PA from VPA to LPA has risen in China, they still exhibit sedentary behavior, with an average of 6.37 h sitting per day, and 17.48% of students sitting more than 8 h per day. This is consistent with the results for other Chinese students (27, 28). Wallace et al. (48) revealed that a sedentary lifestyle promotes the development of KOA by increasing the severity of the disease, according to animal experiments. Multiple pathways play a role, such as promoting the growth of functionally deficient joint tissues and the accumulation of excess body weight (48). Therefore, counteracting sedentary behavior among college students is essential for enhancing knee health, and physical activity load should be adapted to the joint based on the Physical Stress Theory. Hence, studies on adaptive joint physical activity and targeted interventions should be developed in future.

Although our study has implications for future research, several limitations should be noted. First, although the sample size was relatively large, some bias might exist due to using convenience sampling and snowball-style sharing of questionnaire links, including potential influence on findings from overrepresentation of physically active students, high proportion of underweight participants, and self-selection bias, as well as regional limitations (mainly Zhejiang and Beijing), which might limit the generalizability of this study. Second, the results of this study did not confirm the causality of the relationships between knee symptoms and PA or other independent variables among college students owing to the cross-sectional design. Third, although previous studies have confirmed the reliability and validity of the single self-reported item and have demonstrated a strong correlation between knee symptoms and radiographic KOA (11–13), potential misclassification and information bias might exist due to limited construct validity in this study, especially in the absence of X-rays or other imaging examinations to definitively confirm the physiological structure of the joints. To some extent, this might be biased when extending the results to KOA. In addition, due to the lack of reported data on knee symptoms among college students, this study compared its findings to existing evidence derived from middle-aged or older populations with KOA or knee problems. Extrapolating findings from self-reported symptoms in college students to future KOA risk has limited applicability because the biological mechanisms, activity patterns, and disease stages differ substantially between these populations. Finally, given that knee symptoms, PA, and other variables are self-reported, the potential for recall bias and reporting bias exists. Of note, the PA scoring system employed in this study remains unvalidated against standard instruments (e.g., IPAQ), which may compromise the comparability of our findings. Although we confirmed the association between knee symptoms and PA as well as several other factors, residual confounding due to unassessed variables (e.g., biomechanical characteristics, sports history, and psychological traits) cannot be ruled out and warrants further investigation.

## Conclusion

This study suggests that maintaining knee health among college students is crucial, and tailored interventions focusing on guided physical activity according to the Physical Stress Theory should be developed. This may be beneficial in decreasing the odds of knee symptoms and further structural damage.

## Data Availability

The raw data supporting the conclusions of this article will be made available by the authors, without undue reservation.
