# Supplementary material for: Knee symptoms among college students and their relationship with physical activity behaviors: a cross-sectional survey
Source: Front Public Health. 2026 Jul 22;14:1879925. doi: 10.3389/fpubh.2026.1879925 (PMC13437316; doi:10.3389/fpubh.2026.1879925)
Supplement: Supplementary file 1 [file Supplementary_Table.DOCX]

**Results of sensitivity analysis for PA sore system**

We conducted sensitivity analyses of the PA score system using alternative categorizations with lower or higher cutoff values for the moderate‑to‑high level. In Model 1, PA scores were classified as follows: no PA (0 points), low PA (1–2 points), and moderate‑to‑high PA (3–12 points). In Model 2, PA scores were classified as: no PA (0 points), low PA (1–6 points), and moderate‑to‑high PA (7–12 points). Although similar significant results were observed in both models (as presented in Supplementary Table 1), the model fit indices indicated that the present cutoff values provided a better fit.

Supplementary Table 1. Sensitivity analysis for PA score system using the multivariable logistic regression models with OR (95% CI) of knee symptoms

| **Predictors** | **Present model** | **Model1** | **Model2** |
| --- | --- | --- | --- |
| Age (years) | 1.08 (1.01–1.15) * | 1.08 (1.01-1.16) * | 1.08 (1.02-1.15) * |
| Female | — | — | — |
| BMI level |  |  |  |
| Normal weight | Reference | Reference | Reference |
| Under weight | 1.14 (0.75–1.75) | 1.09 (0.71-1.68) | 1.11 (0.72-1.71) |
| Overweight | 1.02 (0.55–1.90) | 0.95 (0.53-1.71) | 0.93 (0.50-1.72) |
| Obese | 0.93 (0.36–2.39) | 0.94 (0.39-2.29) | 0.95 (0.41-2.20) |
| Knee sounds | 3.54 (2.43–5.16) *** | 3.73 (2.60-5.34) *** | 3.77 (2.59-5.47) *** |
| Knee injury | 7.13 (4.30–11.82) *** | 8.00 (5.01-12.78) *** | 8.37 (5.27-13.28) *** |
| Steps per day |  |  |  |
| <3000 | — | — | — |
| 3000~4999 | — | — | — |
| 5000~6999 | — | — | — |
| 7000~10000 | — | — | — |
| >10000 | — | — | — |
| Climbing floors per day |  |  |  |
| <5 | Reference | Reference | Reference |
| 6~10 | 1.03 (0.67–1.60) | 1.10 (0.72-1.69) | 1.09 (0.72-1.67) |
| 11~15 | 1.23 (0.64–2.36) | 1.20 (0.60-2.39) | 1.25 (0.62-2.51) |
| 16~20 | 1.26 (0.64–2.49) | 1.35 (0.67-2.70) | 1.29 (0.65-2.54) |
| >20 | 2.37 (1.06–5.29) * | 2.27 (1.02-5.06) * | 2.28 (1.02-5.07) * |
| Sitting hours per day (h) |  |  |  |
| <5 | — | — | — |
| 5~8 | — | — | — |
| 8~10 | — | — | — |
| >10 | — | — | — |
| Physical activity score ^§^ |  |  |  |
| VPA score |  |  |  |
| Low level | 1.31 (0.85–2.01) | 1.45 (0.91-2.32) | 1.43 (0.95-2.16) |
| Middle-to-high level | 2.66 (1.40–5.06) *** | 1.75 (1.10-2.78) * | 5.30 (2.15-13.02) *** |
| MPA score |  |  |  |
| Low level | 0.82 (0.49–1.38) | 0.76 (0.46-1.24) | 0.77 (0.49-1.22) |
| Middle-to-high level | 0.74 (0.34–1.59) | 0.74 (0.44-1.26) | 0.61 (0.24-1.58) |
| LPA score |  |  |  |
| Low level |  | — | — |
| Middle-to-high level |  | — | — |

*P≤0.05, **P<0.01, ***P<0.001; § For PA scores analysis, no PA was set as reference.

Abbreviation: BMI = Body mass index; CI = Confidence interval; LPA = Light physical activity; MPA = Moderate physical activity; OR= Odds ratio; PA = Physical activity; VPA = Vigorous physical activity.

Supplementary Table 2. Model fit indices for multivariable logistic regression models

| Indices | Present model | Model 1 | Model 2 |
| --- | --- | --- | --- |
| Hosmer-Lemeshow χ^2^ | 6.27 | 9.73 | 10.2 |
| p-value | 0.62 | 0.88 | 0.25 |
| AIC | 845.72 | 859.22 | 850.52 |
| BIC | 930.63 | 934.33 | 925.64 |
| Nagelkerke pseudo-R^2^ | 0.17 | 0.19 | 0.20 |

Abbreviation: AIC: Akaike information criterion; BIC: Bayesian information criterion.
